# Interleukin-15 Plays a Central Role in Human Kidney Physiology and Cancer through the γc Signaling Pathway

**DOI:** 10.1371/journal.pone.0031624

**Published:** 2012-02-21

**Authors:** Julien Giron-Michel, Sandy Azzi, Krystel Khawam, Erwan Mortier, Anne Caignard, Aurore Devocelle, Silvano Ferrini, Michela Croce, Hélène François, Lola Lecru, Bernard Charpentier, Salem Chouaib, Bruno Azzarone, Pierre Eid

**Affiliations:** 1 INSERM UMR 1014, Hôpital Paul Brousse, Villejuif, France; 2 Université Paris-Sud P11, Paris, France; 3 INSERM UMRS 892, Institut de Recherche Thérapeutique de l'Université de Nantes (IRT UN), Nantes, France; 4 Institut Cochin, Université Paris Descartes, INSERM U1016, Paris, France; 5 Laboratory of Immunotherapy, Instituto Nazionale per la Ricerca sul Cancro, Genova, Italy; 6 INSERM UMR 753, Université de Paris-Sud, Institut Gustave Roussy (IGR), Villejuif, France; Institut Pasteur, France

## Abstract

The ability of Interleukin-15 (IL-15) to activate many immune antitumor mechanisms renders the cytokine a good candidate for the therapy of solid tumors, particularly renal cell carcinoma. Although IL-15 is being currently used in clinical trials, the function of the cytokine on kidney's components has not been extensively studied; we thus investigated the role of IL-15 on normal and tumor renal epithelial cells. Herein, we analyzed the expression and the biological functions of IL-15 in normal renal proximal tubuli (RPTEC) and in their neoplastic counterparts, the renal clear cell carcinomas (RCC). This study shows that RPTEC express a functional heterotrimeric IL-15Rαβγc complex whose stimulation with physiologic concentrations of rhIL-15 is sufficient to inhibit epithelial mesenchymal transition (EMT) commitment preserving E-cadherin expression. Indeed, IL-15 is not only a survival factor for epithelial cells, but it can also preserve the renal epithelial phenotype through the γc-signaling pathway, demonstrating that the cytokine possess a wide range of action in epithelial homeostasis. In contrast, in RCC *in vitro* and *in vivo* studies reveal a defect in the expression of γc-receptor and JAK3 associated kinase, which strongly impacts IL-15 signaling. Indeed, in the absence of the γc/JAK3 couple we demonstrate the assembly of an unprecedented functional high affinity IL-15Rαβ heterodimer, that in response to physiologic concentrations of IL-15, triggers an unbalanced signal causing the down-regulation of the tumor suppressor gene E-cadherin, favoring RCC EMT process. Remarkably, the rescue of IL-15/γc-dependent signaling (STAT5), by co-transfecting γc and JAK3 in RCC, inhibits EMT reversion. In conclusion, these data highlight the central role of IL-15 and γc-receptor signaling in renal homeostasis through the control of E-cadherin expression and preservation of epithelial phenotype both in RPTEC (up-regulation) and RCC (down-regulation).

## Introduction

Interleukin-15 (IL-15) is a pleiotropic cytokine involved in innate immunity as well as in functions outside the immune system. IL-15 functional diversity is explained in part by its complex mechanisms of action [1], [2] involving not only soluble and membrane forms of the cytokine but also different IL-15 receptors (IL-15R) with specific affinities and signal transduction pathways [3], [4]. Indeed, IL-15 binds to the IL-15Rα private chain with high affinity (Kd≥10^−11^ M), which deliver a specific signaling in response to IL-15 (NF-κB, Syk). IL-15 shares with IL-2 the IL-2Rβ (CD122) and IL-2Rγ (CD132, γc) subunits, which form either a functional receptor of high (IL-15Rαβγ, Kd≥10^−11^ M) or intermediate affinity (IL-15Rβγ, Kd = 4 nM) for IL-15, allowing signaling through a cascade that involves the JAK/STAT, MAPK and PI3-K transduction pathways.

The common γc receptor chain is a key component of the four-helix-bundle cytokines family, allowing through its association with the Janus tyrosine kinase 3 (JAK3), the activation of STAT molecules (Signal Transducers and Activators of Transcription) [5]. JAK3 phosphorylates different downstream STATs, in relationship to the type of the receptor complex involved. Thus, IL-4 predominantly signals through STAT-6, whereas IL-2, IL-7, IL-9 and IL-21 act through STAT-1 and STAT-3 and IL-15 mainly activates STAT-5 [6], [7], [8]. Both the γc and JAK3 are essential for the function of all the cytokine receptors of this family and are required for the development of the lymphoid cell system. Indeed, genetic defects of γc or JAK3 results in a severe combined immune deficiency (SCID) characterized by the lack of T, B and NK cells in both mice and humans [9], [10], [11]. However, it must be stated that in SCID patients, the corrective gene therapy for γc can act as a contributor to genesis of cell lymphomas [9], [11]. The IL-2Rγ chain is also expressed in non-lymphoid cells and is detected for instance on certain tumoral epithelial cells [12], [13], [14] where the amount of γc is involved in the mechanisms that govern the cell growth. IL-2Rγ is also found in normal epithelial cells where it modulates signal transduction of different members of the γc family even if its specific biological functions have not yet been clearly defined [13], [15], [16].

The human epithelial cells of various tissues produce IL-15, which acts not only on immune cells (e.g., IELs in the gut), but also on epithelial cells, mainly via its anti-apoptotic action [17], [18], [19], [20]. Thus, it was shown that human and mouse renal tubular epithelial cells (RPTEC) constitutively express the receptor IL-15Rαβγ [15] and secrete the cytokine IL-15 [21], which plays an important role in renal physiology as an autocrine survival factor. Indeed, increased sensitivity of cells to apoptosis is observed in the damaged kidney of IL-15−/−, IL-15Rα−/− knockout mice or during acute renal injury induced by different protocols that induce a decrease in IL-15 production by epithelial cells [20], [22]. IL-15 enhances intestinal barrier function by promoting the formation of tight junctions between epithelial cells [23]. These results suggest that the IL-15 survival factor may have other functions, that remain to be explored in renal epithelial cells [15], [24], [25], [26].

Due to its immuno-stimulating activity in several preclinical models, the use of IL-15 could be a useful cytokine for the treatment of kidney cancers. Although IL-15 is currently being tested in clinical trials for the treatment of kidney cancer (NCT01021059 Protocol) [2], the functions of the cytokine on normal epithelial cells as well as tumor cells remain poorly studied. In order to better understand the functions of the cytokine, we propose to study the IL-15/IL15R system on human kidney epithelial cells of normal origin (RPTEC) and on cells of tumor origin (RCC).

Our data show that both *in vitro and in vivo* primary normal renal proximal tubular cells (RPTEC) express the IL-15Rαβγ receptor, whereas expression of the γc chain and JAK3 is severely impaired in renal clear cancer cells (RCC). The differential expression of γc chain and JAK3 has a marked impact on renal homeostasis since soluble IL-15 in RPTEC through the γc chain signaling pathway preserves the expression of the tumor suppressor gene E-cadherin, inhibiting their epithelial-mesenchymal transition (EMT) commitment. By contrast, loss of the γc chain and JAK3 in primary RCC leads to the formation of an unprecedented functional IL-15Rαβ high affinity heterodimer, whose stimulation with soluble IL-15 causes the down-regulation of the E-cadherin expression favoring the EMT process.

## Materials and Methods

### Antibodies, Cytokines, and Reagents

Antibodies (Abs) against IL-15 (L-20), IL-15Rα (sc-9172), IL-2Rβ (sc-1046), IL-2Rγ (sc-670), JAK3 (sc-513), and vimentin (sc-73260) were obtained from Santa Cruz Biotechnology (Delaware, CA). Antibodies against phosphorylated ERK (4377), phosphorylated IκB (4921), STAT5 (9358) phosphorylated STAT5 (9356), and the Alexa fluor-conjugated rabbit monoclonal antibody against phosphorylated STAT5 (3939) were obtained from Cell Signaling (Beverly, MA). Antibodies against IL-15Rα (AF247), E-cadherin (AF648) and PE-conjugated anti-E-cadherin (FAB18381P) were obtained from R&D Systems Europe Ltd (Abingdon, Oxon, U.K.), as well as neutralizing anti-IL-2Rγ (mAb2842) mAb. The FITC-conjugated anti-fibroblast ASO2 was from *Dianova* GmbH (Hamburg, Germany) and the pan-cytokeratin (CK) Ab from EXBIO (Prague, Czech Republic). Rhodamine-conjugated phalloidin for F-actin detection and zonula occludens-1 (ZO-1) were obtained from Invitrogen (Cergy Pontoise, France). Antibody against JAK3 (07-1488) was from Millipore (Saint-Quentin-en-Yvelines, France) and anti-IL-2Rβ (AB364) was from Assay Biotech (Interchim BioScience Innovations, France). The anti-β-actin mAb (A1978) was from Sigma-Aldrich (St. Louis, MO, USA). Recombinant human non-glycosylated IL-15 (rhIL-15) and neutralizing anti-IL-2Rβ Mikβ1 mAb were obtained from ImmunoTools (Friesoythe, Germany) and Horseradish peroxidase (HRP)-conjugated and fluorescent-conjugated secondary Abs were from Jackson ImmunoResearch. JAK3 inhibitor (CP-690, 550) and STAT5 inhibitor (573108) were purchased from Calbiochem (SD, CA). Anti-IL-15Rα M161 mAb was provided by Amgen (Thousand Oaks, CA).

### Primary cells and cell lines

Primary human normal Renal Proximal Tubular Epithelial Cell (RPTEC) derived from a non-cancerous kidney (Lonza, Verviers, Belgium) and expanded *in vitro* following manufacturer's instructions. REGM culture medium of RPTEC was daily changed to maintain epithelial characteristics. Primary tumor cells were obtained by enzymatic digestion of fragments of clear cell renal carcinomas (RCC) as described previously [27]. Subsequently, the digested cellular suspensions were seeded onto plastic Petri dishes using RPMI 1640 supplemented with 10% fetal calf serum, 1% MEM sodium pyruvate, 1% penicillin/streptomycin (Life Technologies). In these culture conditions, only a fraction of cells adheres to the plastic surface and proliferates, generating RCC primary cultures and subsequently cell lines (RCC5, RCC7, RCC8).

The human kidney carcinoma ACHN (ATCC, CRL-1611), MCF-7 (human breast cancer cells) and U937 (human monocytic leukemia cells) cell lines were cultivated as described above. The erythroleukemia cell line TF1β was maintained in RPMI 1640 medium supplemented with 5 ng/ml GM-CSF and 250 µg/ml geneticin G418. Peripheral Blood Lymphocytes (PBL) were prepared as previously described [28]. Human samples were collected and handled in the full respect of the declaration of Helsinki.

#### Reverse-transcription (RT)-PCR analysis

Reverse-transcription (RT)-PCR analysis was performed as previously described [29]. Specific RT-PCR primers are detailed below.

IL-15Rα F 5′-GGCGACGCGGGGCATCAC; R 5′-TGCCTGTGGCCCTGTGGATA; IL-2Rβ F 5′-GAATTCCCTGGAGAGATGGCCACGGTCCCA; R 5′-GAATTCGAGGTTTG GAAATGGATGGACCAAGT; γc chain F 5′-CCAGGACCCACGGGAACCCA; R 5′-GG TGGGAATTCGGGGCATCG; JAK3 F 5′-CGTTCATGCAGCCTCTTGTTC; R 5′-GCGCA CCGTCCTCCGAATAC; β-actin F 5′-GTGGGGCGCCCCAGGCACCA; R 5′-CTCCTTA ATGTCACGCACGATTTC.

### IL-15 binding assays

Human rIL-15 was radiolabeled with iodine (specific radioactivity approximately 2000 cpm/fmol) using a chloramine-T method and binding experiments were performed as described previously [30]. Nonspecific binding was determined in the presence of 100-fold excess of unlabeled cytokine. For the IL-15 binding experiments, RCC7 cells were incubated with increasing concentrations of labeled rIL-15. Regression analysis of the binding data was accomplished using a one-site equilibrium binding equation (Grafit, Erithacus Software, Staines, UK), and data were plotted in the Scatchard coordinate system. For inhibition of IL-15 binding experiments, RCC7 cells were incubated, in the presence of increased concentrations of iodinated rIL-15, and fixed concentrations of neutralizing antibodies against IL-2Rβ (Mikβ1, 10 µg/ml) or IL-2Rγ (mAB2842, 1 µg/ml) chains. Regression analysis of data was accomplished using a 4-parameter logistic equation (Grafit, Erithacus Software).

### Plasmids and transient transfection

pCMV6 vector encoding full-length cDNA Myc-DDK-tagged ORF of human interleukin 2 receptor gamma (IL2Rγ) was purchased from Origene Technologies Inc (Rockville, MD, USA) and full length human JAK3 cDNA subcloned between the EcoRI-XhoI restriction sites of the *pcDNA3.1* eukaryotic expression vector was a kind gift from Dr. Franck Gesbert (UMR1004, Inserm, France). Plasmids were transformed into Top 10 competent bacteria cells according to the manufacturer's protocol (Invitrogen, Carlsbad, CA), extracted using a Maxiprep kit (Qiagen, Valencia, CA), and amplified by culture in Luria-Bertani-ampicillin broth. cDNAs were transiently transfected into cells according to manufacturer's instructions. Briefly, cells were plated into six-well plates (0.25×10^6^ cells/well) and cultured overnight in complete medium. The transient mixture, which contained 1.0 µg of plasmid DNA and 6 µl of Fugene 6 transfection reagent (Roche Diagnostics, Indianapolis, IN) in 100 µl of serum-free DMEM medium (Invitrogen), was mixed for 20 min at room temperature and then added to each well with complete medium for 48 h. The empty *pcDNA3.1* vector was transfected as control.

### Flow Cytometry Analyses

For all assays described below, we acquired fluorescence data for 10,000 cells on a FACScalibur flow cytometer (BD Biosciences) and the data was analyzed using CellQuest software (BD Biosciences). Three replicates were used for each condition and the experiment was repeated at least three times.

#### Expression of Cellular Antigens

Expression of cell surface (E-cadherin) and intracellular (Vimentin, Pan-CK) antigens was analyzed by flow cytometry as previously described [29], [31], [32]. Briefly, suspensions of enzymatically detached cells were permeabilized or not with BD Cytofix/Cytoperm reagent (BD Pharmingen, Le Pont De Claix, France), and 10^5^ cells were suspended in RPMI medium supplemented with 1% FCS and stained with conjugated antibodies directed against the above-mentioned cell markers. Subsequently, cells were fixed by incubation with 1% paraformaldehyde in phosphate-buffered saline (PBS) for 20 minutes at room temperature and analyzed by flow cytometry.

#### STAT5 Activation

We investigated STAT5 activation in RCCWT (RCC wild type) and IL-2Rγ and/or JAK3-transfected RCC by treating cells with 10 pg/mL of rhIL-15 during 40 minutes. Treated and untreated cells were detached by trypsin, washed, and fixed by incubation with 1% paraformaldehyde in PBS for 20 minutes at room temperature. The cells were permeabilized by resuspension, with vortexing, in ice-cold methanol and incubated at 4°C for 10 minutes. The cells were washed in 1% BSA in PBS and incubated with an Alexa Fluor 488–conjugated mouse monoclonal antibody against phosphorylated STAT5 for 60 minutes at 4°C.

### Immunoprecipitation and immunoblot Analyses

All immunoblotting (WB) were performed as previously described [29]. For immunoprecipitation, PBS-washed cell pellet was lysed in 1 ml 1% NP-40 and 0.1% SDS, 50 mM sodium phosphate buffer pH 7.8, 150 mM NaCl, 1 mM sodium orthovanadate, 1 mM EDTA, 1 mM EGTA, 1 mM AEBSF, aprotinin, leupeptin and pepstatin (5 µg/ml each). After 15 min shaking at 4°C, the suspension was centrifuged (30 min at 14,000 rpm, 4°C). The supernatant was added to 20 µl of Sepharose-conjugated-M161 (anti-IL-15Rα, 2 µg/µl). After 4 h agitation at 4°C, the immune complexes were washed 5 times with 1 ml of lysis buffer and applied on 10% PAGE-SDS. Blots were processed as previously described [29].

### Immunocytochemistry

Cells were dispensed into eight-well compartments of Lab-Tek tissue culture chamber slides (1×10^5^ cells per well; Nunc, Naperville, Ill.) and at confluence, treated or not with 10 pg/ml of rhIL-15 for 5 days. For membrane staining, cells were fixed with cold methanol∶acetone (1∶1) at −20°C for 10 min, washed then blocked with PBS 3% BSA for 60 min. Cells were incubated with anti-human E-cadherin or FITC-conjugated anti-ASO2 antibodies overnight at 4°C. Subsequently, cells were washed, incubated for 30 min with an AlexaFluor488-conjugated rabbit anti-goat antibody. For intracellular staining, the cells were fixed with 4% (wt/vol) paraformaldehyde in PBS and permeabilized by incubation for 1 minute with 0.5% Triton X-100 in PBS. The cells were incubated with blocking solution (3% BSA in PBS) and incubated overnight at 4°C with the various antibodies. The cells were then washed and incubated with Alexa Fluor 488–conjugated rabbit anti-mouse or anti-goat IgG diluted in blocking solution and incubated for 30 minutes. F-actin organization was revealed staining the cells with 0.2 µg/mL of rhodamine-conjugated phalloidin for 20 minutes. The cells were washed with PBS, mounted in 4,6-diamidino-2-phenylindole (DAPI, Invitrogen, Cergy Pontoise, France), and visualized by fluorescence microscopy (Leica, Germany).

### Immunohistochemistry on Paraffin-Embedded renal tumor and normal samples

Biopsies from 3 normal and 10 tumor sections from nephrectomized kidneys with renal cell carcinoma were sectioned at 4 µm onto Superfrost plus slides. Deparaffinized slides were rehydrated in graded alcohols, and subjected to heat-induced epitope retrieval by immersing them in 0.01 mol/L citrate buffer (pH 6.0). Sections were incubated overnight at 4°C with anti-IL-2Rβ (AB364), anti-IL-2Rγ (sc-670) or anti-JAK3 (07-1488) Abs, PBS-rinsed and incubated with HRP-secondary Ab for 45 min. Analysis was performed by standard methods using diaminobenzidine after counterstaining the sections with hematoxylin. The negative control was subjected to all treatments omitting primary antibody. Slides were scanned using an Aperio scanner (Vista, CA) and staining was quantified using a morphometric TRIBVN software (Montrouge, France).

## Results

### IL-15R expression in RPTEC and RCC primary cultures

In order to shed light on the function of IL-15 in the renal human model, we investigated the expression of IL-15 receptor subunits (IL-15Rαβγ) on primary cultures of normal Renal Proximal Tubular Epithelial Cell (RPTEC) and clear cell renal carcinomas (RCC).

**Figure 1:**
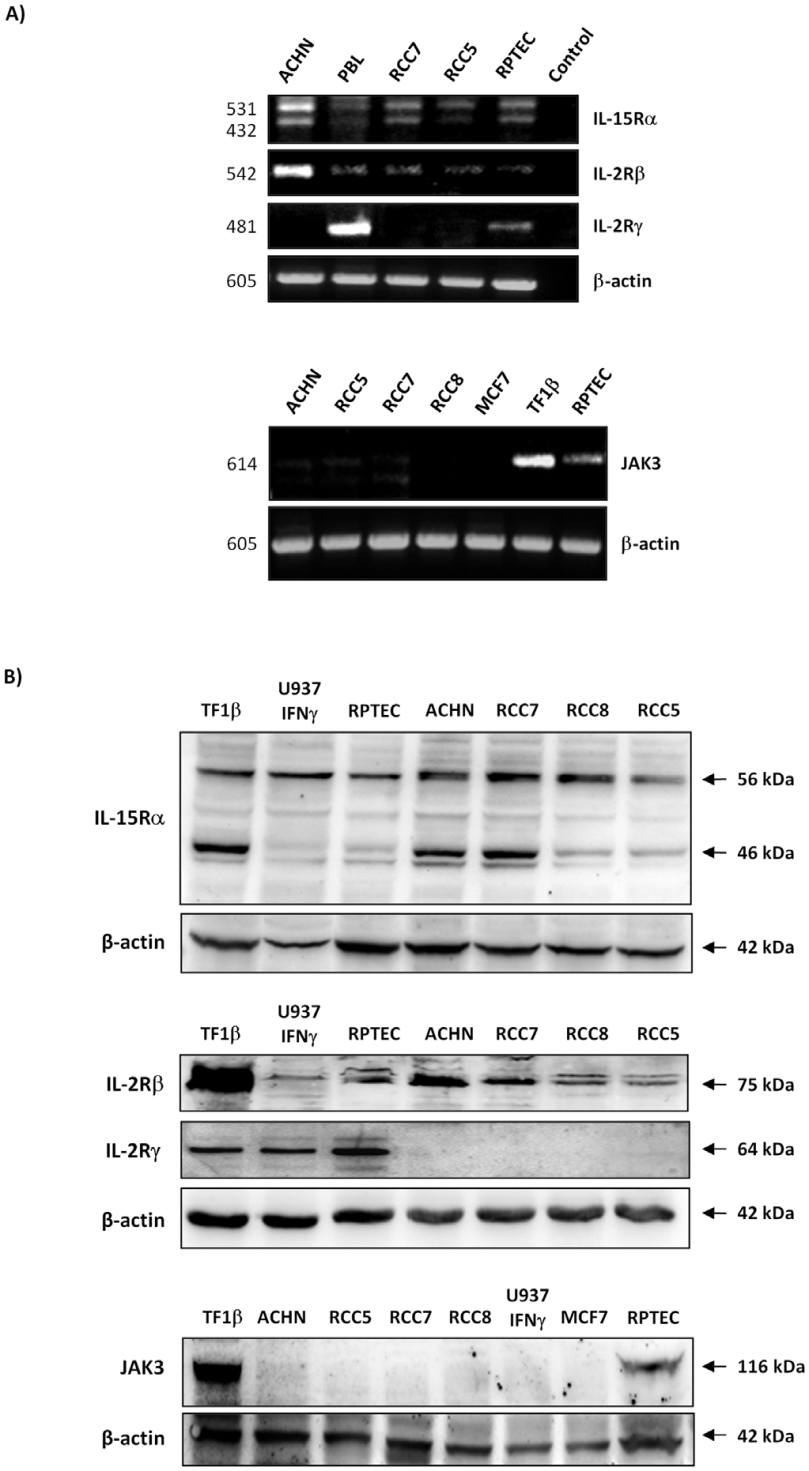
Normal and tumoral renal epithelial cells express different IL-15R subtypes. Analysis of IL-15R and JAK3 expression was performed by RT-PCR (**A**) and immunoblotting (**B**) on primary normal (RPTEC) and tumoral (RCC5, RCC7, RCC8) epithelial cells and the ACHN cell line. Data show that RPTEC express the three chains of the IL-15R (αβγ) and JAK3 whereas γc and JAK3 proteins were not detected in RCC. Specific primers or Abs against IL-15Rα (AF247), IL-2Rβ (sc-1046), IL-2Rγ (sc-670) and JAK3 (sc-513) were used. PBL, TF1β, MCF7 and IFNγ-activated U937 cells were used as controls. Housekeeping β-actin was used as loading control.

RT-PCR analysis (Figure 1A, upper panel) shows that RPTEC, in agreement with the positive PBL control, express different transcripts for the IL-15Rα chain (432 and 531 bp), the transcript for the IL-15Rβ (542 bp) and the transcript for the γc chain (480 bp). In contrast, only the IL-15Rα and β chains, but not the γc chain, were detected either on RCC (RCC5 and RCC7) or ACHN cell line. Since JAK3 kinase specifically interacts with its cognate receptor γc chain, and expression of both molecules is interdependent [33], we further analyzed, by RT-PCR, JAK3 expression in normal and tumor renal cells (Figure 1A, lower panel). JAK3 kinase was detected in the positive haematopoietic control cell line TF1β and RPTEC, while a weak messenger amount or absent (RCC8) was detected in RCC analysed. No JAK3 messenger was detected in the MCF-7 control cell [34].

To confirm the differential expression of the receptor subunits and the JAK3 kinase at the protein level, immunoblotting was performed on both normal and tumoral cells. The analysis confirmed that RPTEC, RCC and TF1β cells express two major bands of 46 and 56 kDa specific for the IL-15Rα (Figure 1B, upper panel) and a 75 kDa band for the IL-15Rβ chain (Figure 1B, middle panel). The absence of the γc chain in RCC was confirmed since the 64 kDa band is detected exclusively in positive control cell lines (TF1β and IFN-γ treated U937) and RPTEC (Figure 1B, middle panel). JAK3 molecule (116 kDa) was detected in TF1β and RPTEC cells, whereas immunoblotting did not detect the kinase in RCC, as well as in MCF-7 and IFN-γ treated U937 control cell lines as previously reported [34], [35] (Figure 1B, lower panel). In all above-mentioned experiments, we also studied renal ATCC-CRL-1611 cell line (ACHN) that display IL-15R and JAK3 expression homologous to those observed in RCC primary samples.

### Loss of γc chain in renal clear cell carcinoma tissues samples

**Figure 2:**
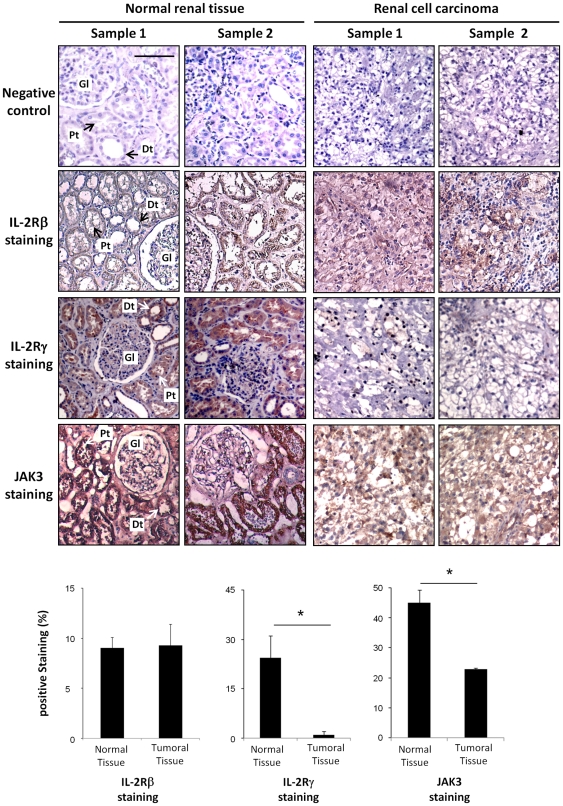
Immunohistochemical staining for IL-2Rβ, IL-2Rγ and JAK3 in normal and neoplastic kidney specimens. Hematoxylin staining of biopsies from 2 normal samples reveals the presence of the normal kidney structures (glomerulus (Gl), distal (Dt) and proximal (Pt) tubules), while analysis of two different cancer specimens shows that the normal tissue architecture is totally lost and are replaced by tumor cells with clear cell morphology, characterized by optically clear cytoplasm and sharply outlined cell membrane. Whereas no difference on IL-2Rβ is observed between normal and tumoral tissue samples, the IL-2Rγ staining, localized to both proximal and distal tubuli in normal tissue samples, is not found in the tumor samples. A strong JAK3 staining is localized to both proximal and distal tubular cells of normal tissues, while a very faint JAK3 protein expression is detected in tumor samples. Two representative samples of a total of ten are shown for each staining. Negative control was subjected to all treatments omitting primary antibody. Scale bars, 50 µm. Staining was quantified using a morphometric TRIBVN software (Montrouge, France) and results are presented as histograms.

In order to confirm our *in vitro* data, IL-2Rβ chain, γc chain and JAK3 immunohistochemical stainings were performed on normal and tumor sections of nephrectomized kidneys with renal cell carcinoma. Hematoxylin staining of paraffin embedded human kidney sections revealed under light microscopy the presence of glomeruli (Gl) and several distal (Dt) and proximal (Pt) tubules in the normal tissue samples (Figure 2). By contrast, these kidney structures are no longer present in the renal carcinoma section, showing tumor cells with clear cell morphology, characterized by optically clear cytoplasm and sharply outlined cell membrane.

Immunohistochemical staining on two different normal renal specimens reveals that the IL-2Rβ chain, γc chain and a strong JAK3 expression are detected on proximal and distal tubular cells. By contrast, analysis of different tumor samples revealed the absence of γc chain staining (P<0.01) with a very faint JAK3 protein expression (P<0.01) while, no significant differences (P>0.05) in the expression of the IL-2Rβ chain were observed between normal and tumoral tissues therefore confirming the results obtained *in vitro* in primary cultures of normal and cancer cells.

### Soluble IL-15 triggers a differential cell signal in RCC and RPTEC

To our knowledge, the IL-15Rαβ heterodimer was only described in IL-2Rγ−/− knockout mice, that exhibits an efficient binding and endocytosis of radiolabeled IL-15 [36]. However, the authors did not investigate whether the IL-15Rαβ heterodimer exists as a preformed complex or is formed following IL-15 binding thereby generating a functional heterodimer.

**Figure 3:**
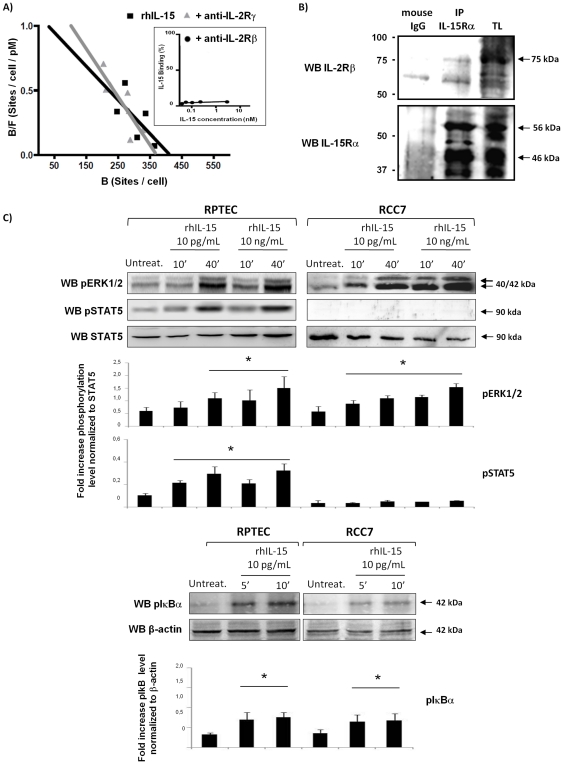
Soluble IL-15 triggers a differential cell signal in RCC and RPTEC. **A) Scatchard's plot analysis: Effects of anti-IL-15Rβ and γc mAbs on IL-15 binding to RCC.** For the IL-15 binding experiments, RCC7 cells were incubated with increasing concentrations of radioiodinated rIL-15 in presence or not of the following neutralizing mAbs: anti-IL-2Rβ and IL-2Rγ. The nonspecific cell binding was determined in the presence of radioiodinated rhIL-15 and a 100-fold excess of unlabeled rhIL-15. Cell-bound (B) and unbound (free, F) fractions were measured, and the specific bound fraction was calculated by subtracting the nonspecific binding from the cell-bound fraction. On the ordinate is plotted the ratio of the specific bound fraction (expressed in sites per cell) over the total concentration (bound plus free) of radioiodinated rIL-15 (expressed in pM). On the abscissa bound fraction (expressed in sites per cell). The high affinity specific IL-15 binding (Kd = 375 pM, 413 IL-15 binding sites per cell), which was completely abrogated by neutralizing antibody against the IL-2Rβ (inset) but not the γc chain, suggested the presence on RCC of an IL-15Rα/IL-2Rβ complex. **B**) Detection of IL-15Rαβ complex by immunoprecipitation (IP) with anti-IL-15Rα (M161) or mouse IgG protein G-Sepharose-conjugate on total lysate (TL) of RCC7. Immunoprecipitated complexes were blotted either with anti-IL-2Rβ (sc-1046) and anti-IL-15Rα (sc-9172). **C**) Stimulation for 10 and 40 min with physiologic (10 pg/mL) and supra-physiologic (10 ng/mL) concentrations of rhIL-15 induces the phosphorylation of MAPK ERK1/2 and IκBα in RPTEC and RCC7, whereas STAT5 activation was only observed in RPTEC. Histograms represent densitometry comparison of each factor normalized to β-actin in 3 different RCC (RCC5, RCC7, RCC8) and 3 RPTEC batches. * P<0.05 versus control, Mann-Whitney test. One experiment representative of a total of three is shown.

To evaluate IL-15 binding on γc-negative RCC, we first analyzed radiolabeled recombinant human IL-15 (rhIL-15) binding to RCC7 cells by Scatchard's plot analysis (Figure 3A). The data reveals the presence of a single class of high affinity receptors (Kd = 375 pM, 413 IL-15 binding sites per cell). Specific IL-15 binding was completely abrogated by the anti-IL-2Rβ mAb Mikβ1 (Figure 3A, inset), while neutralizing anti-IL-2Rγ mAb had no effect on specific IL-15 binding, suggesting that the binding indeed reflected the presence of an IL-15Rα/IL-2Rβ complex.

To confirm the presence of an IL-15Rα/IL-2Rβ complex heterodimer, the potential interactions between the IL-15Rα and IL-2Rβ chains in RCC were investigated performing co-immunoprecipitation experiments on RCC7 cell lysates by immunoadsorption to Sepharose-conjugated anti-IL-15Rα (M161) (Figure 3B). Anti-IL-2Rβ immunoblotting reveals the presence of a specific 75 kDa protein (upper panel) while anti-IL-15Rα blot, performed on the same membrane, shows the expression of specific bands of 56 and 46 kDa (lower panel) indicating that IL-2Rβ and IL-15Rα receptor subunits are constitutively associated, forming an IL-15Rαβ heterodimer in the absence of the cytokine.

In order to determine whether the IL-15Rαβ complex expressed on RCC is functional, we investigated signal transduction activation in normal and tumor renal cells treated with physiologic (10 pg/mL) and supra-physiologic (10 ng/mL) concentrations of rhIL-15 (Figure 3C). Stimulation with rhIL-15 (10–40 min) induced in RCC7 the phosphorylation of the MAPK ERK1/2 at both concentrations, while no STAT5 phosphorylation was observed even in the presence of 10 ng/mL rhIL-15 (Figure 3B, upper panel). In contrast, in RPTEC expressing the heterotrimeric receptor complex, the activation of MAPK ERK1/2 and STAT5 was induced in response to 10 pg/mL and 10 ng/mL of rhIL-15. Moreover, there is a rapid phosphorylation of IκBα, a key event in the activation of the transcription factor NF-κB, in RPTEC and RCC7 in response to physiologic rhIL-15 concentration (Figure 3B, lower panel), indicating that the IL-15Rαβ complex binds IL-15 at high affinity and is functional.

### Soluble IL-15 controls E-cadherin expression on renal epithelial cells

**Figure 4:**
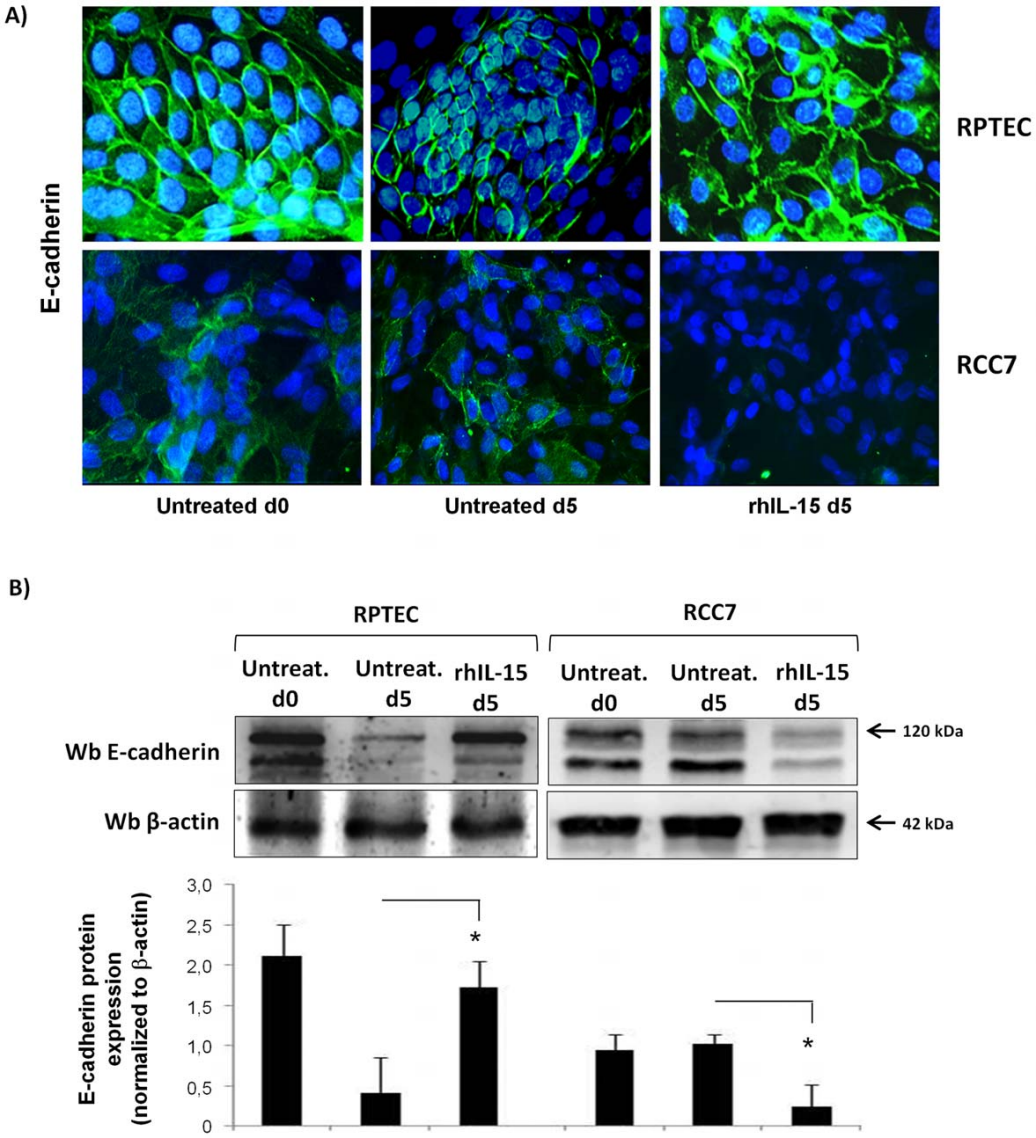
Soluble IL-15, at physiologic concentration, differently controls E-cadherin expression in RCC and RPTEC. Immunofluorescence analysis (**A**) and immunoblot (**B**) show that 5 days rhIL-15 treatment (10 pg/mL) preserves membrane E-cadherin expression on primary normal epithelial cells RPTEC, whereas it induces its down-regulation on RCC7. The medium culture of RPTEC was not changed in order to induce the decrease of E-cadherin expression. Treatment with rhIL-15 was renewed at day 3. Histograms represent densitometry comparison of E-cadherin immunoblots normalized to β-actin in 3 different RCC (RCC5, RCC7, RCC8) cells and 3 RPTEC batches. * P<0.05.

E-cadherin is responsible for maintaining interactions of epithelial cells and is frequently down-regulated during tumor progression [37]. Thus, we investigated the effects of rhIL-15 on E-cadherin expression on both RCC7 and RPTEC by immunofluorescence analysis (Figure 4A) and immunoblotting (Figure 4B). To evaluate the effect of rhIL-15 on normal RPTEC, we used a cell model where the deprivation of corticosteroids, that are powerful inducers of E-cadherin [38], together with absence of daily medium renewal leads within five days to the decrease of E-cadherin expression, without affecting cell viability (97%, data not shown). Immunofluorescence analysis (Figure 4A) shows that normal epithelial cells RPTEC in the first passages (p2) display an epithelial-like morphology characterized by a high level of membrane E-cadherin expression (basal d0) in contrast to five days old RPTEC (basal d5) that exhibit low E-cadherin expression in absence of daily medium renewal. Addition of 10 pg/mL of rhIL-15 during five days preserves the initial E-cadherin level and hence an epithelial-like morphology. In contrast to RPTEC, RCC7 at day 0 and day 5 display a weak E-cadherin expression, which disappears after 5 days of rhIL-15 treatment (Figure 4A). Immunoblotting analysis (Figure 4B) clearly shows the opposite effects of rhIL-15 on the expression of the mature 120 kDa E-cadherin form on RPTEC versus RCC (day 5).

**Figure 5:**
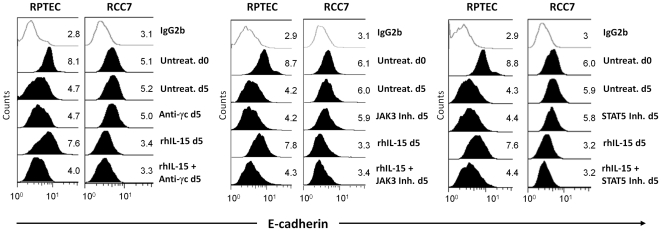
Up-regulation of E-cadherin expression by rhIL-15 on RPTEC is dependent of the βc-dependent signaling pathway. The γc neutralization, as well as JAK3 or STAT5 inhibition, hamper the maintenance of E-cadherin surface expression induced by rhIL-15 on RPTEC without interfering on the E-cadherin down-regulation on rhIL-15-treated RCC7. Cells were pretreated with 1 µg/ml of neutralizing anti-IL2Rγ antibody mAb2842, 0.25 µM of JAK3 inhibitor (CP-690, 550, Calbiochem) or 100 µM of STAT5 inhibitor (STAT5 Inh., 573108, Calbiochem) for 1 h before adding the recombinant cytokine (10 pg/mL) for 5 days. Treatment with rhIL-15 and STAT5 inhibitor was renewed at day 3. White histograms refer to isotype-matched control. Mean fluorescence intensity values for each marker are shown in each histogram. The data are representative of 3 separate experiments performed using different RCC (RCC5, RCC8) and RPTEC batches.

In the light of these data, we asked whether the absence of the γc chain could be involved in the differential modulation of E-cadherin expression by rhIL-15 in RPTEC versus RCC. For this purpose, we used neutralizing anti-γc chain antibody as well as specific inhibitors against JAK3 and STAT5, two molecules involved in the IL-15-induced γc chain signaling. Flow cytometry analysis (Figure 5) showed that the maintenance of E-cadherin by rhIL-15 in RPTEC is counteracted by the neutralizing anti-γc chain antibody (left panel), JAK3 (middle panel) and STAT5 (right panel) specific inhibitors while the different treatments did not interfere with the E-cadherin basal expression on RPTEC. These data strongly support the direct involvement of the IL-2Rγ chain signaling in E-cadherin modulation on renal human epithelial cells. In contrast, anti-γc chain antibody and specific JAK3 and STAT5 inhibitors do not interfere with rhIL-15 induced E-cadherin down-regulation in RCC7. All the above data underline that IL-15 appears to play a major role in the renal homeostasis, regulating E-cadherin expression.

**Figure 6:**
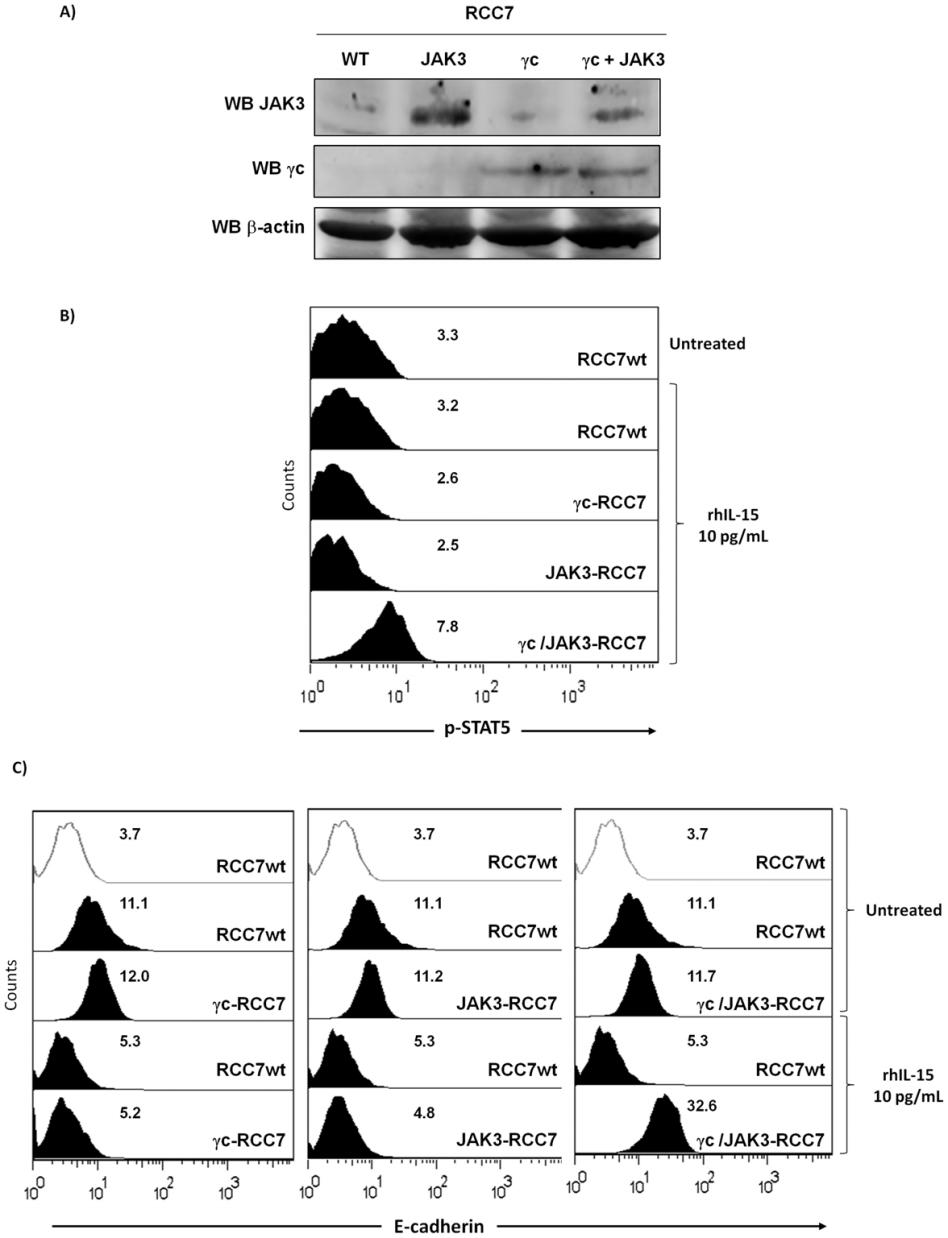
Reestablishment of IL-2Rγ chain-dependent signal transduction pathway in RCC interferes with the rhIL-15-induced E-cadherin down-regulation. RCC7 were transiently transfected for 48 hours with vectors containing IL-2Rγ and/or JAK3 Human cDNA. **A**) Transient expression of IL-2Rγ and JAK3 was analyzed by immunoblotting in each transfected RCC. Immunoblotting for β-actin was used as a control for equal protein loading and transfer. **B**) Flow cytometry shows that 40 min rhIL-15 treatment did not induce STAT5 phosphorylation in IL-2Rγ- or JAK3-transfected RCC while rhIL-15 treatment induced STAT5 phosphorylation in co-transfected cells. **C**) After 48 h, transfected RCC were treated for an additional 48 h with 10 pg/mL of rhIL-15 before evaluating E-cadherin expression by flow cytometry. The introduction of either IL-2Rγ chain, JAK3 or both molecules do not modify E-cadherin expression on untreated rhIL-15 cells, while the E-cadherin down-regulation observed after 48 hours of rhIL-15 treatment was counterbalanced only in co-transfected cells. Mean fluorescence intensity values for each marker are shown in each histogram. One experiment representative of a total of three is shown.

Since rhIL-15 down-regulates E-cadherin expression on RCC lacking IL-2Rγ and JAK3, we asked whether after co-transfection of both molecules it was possible to reestablish IL-15 downstream signaling and subsequently induced E-cadherin expression as observed on RPTEC. RCC7 were transiently transfected for 48 hours with vectors containing IL-2Rγ and/or JAK3 Human cDNA and expression of both molecules was analyzed by immunoblotting (Figure 6A). As expected, the data confirmed that IL-2Rγ chain, JAK3 or both are well expressed after 48 hours in transfected RCC. Flow cytometry (Figure 6B) showed that 40 min rhIL-15 treatment did not induce STAT5 phosphorylation in γc- or JAK3-transfected RCC while rhIL-15 activated STAT5 phosphorylation in co-transfected cells (γc/JAK3-RCC7), suggesting that expression of both molecules is necessary to reestablish the STAT5 signal transduction pathway in RCC. In the light of these results, it was interesting to determine whether the reestablishment of IL-2Rγ chain-dependent signal transduction pathway in RCC could interfere with the rhIL-15-induced E-cadherin down-regulation. The introduction of either IL-2Rγ chain, JAK3 or both molecules did not modify E-cadherin expression on untreated rhIL-15 cells, while the E-cadherin down regulation observed after 48 hours of rhIL-15 treatment was counterbalanced only in co-transfected cells (γc/JAK3-RCC7).

### E-cadherin modulation by soluble IL-15 controls EMT on renal epithelial cells

**Figure 7:**
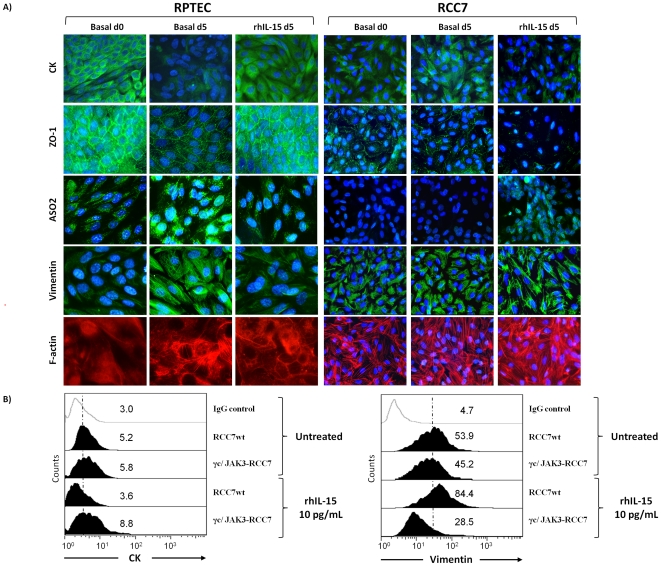
E-cadherin modulation by soluble IL-15 controls epithelial-mesenchymal transition on renal epithelial cells. **A**) Immunofluorescence of cell–cell adhesion molecules show that IL-15 favors epithelial-mesenchymal transition (EMT) on RCC7, whereas it preserves the EMT commitment of RPTEC. The medium culture of RPTEC was not changed in order to induce the EMT process. Cells stimulated or not with 10 pg/ml of rhIL-15 for 5 days, were fixed and stained using standard immunofluorescence procedures with Abs against epithelial (cytokeratins and ZO-1) and mesenchymal markers (F-actin, ASO2 and vimentin). Similar results were obtained using different RCC (RCC5, RCC8) and RPTEC batches. **B**) After 48 h, transfected RCC were treated for an additional 48 h with 10 pg/mL of rhIL-15 before evaluating the epithelial (cytokeratins) and mesenchymal (vimentin) markers expression by flow cytometry. RhIL-15 induced EMT was counterbalanced only in IL-2Rγ/JAK3 co-transfected RCC. Mean fluorescence intensity values for each marker are shown in each histogram. Results are representative of three experiments.

Since down-regulation of E-cadherin, causing the loss of cell-cell adhesions is a key initial step in the process of tubular epithelial-myofibroblast transdifferentiation [39], [40], [41], we asked whether IL-15, which controls E-cadherin expression, could influence this process. Thus, we examined by immunofluorescence the morphology and the cytoskeleton reorganization analyzing several epithelial and mesenchymal molecular markers both on normal RPTEC, deprived of corticosteroids and of daily medium renewal, and on RCC. Deprivation of corticosteroids, together with absence of daily medium change induceed within five days in RPTEC, not only the decrease of E-cadherin expression (Figure 4A), but also an EMT-like phenotype (Figure 7A), characterized by the loss of epithelial markers (cytokeratins (CK) and zonula occludens-1 (ZO-1)) and the acquisition of a fibroblast-like morphology. The mesenchymal phenotype is characterized by the strong expression of the surface fibroblast marker ASO2 [42] and by a cytoskeletal reorganization represented by the formation of large alpha smooth muscle actin (α-SMA) stress fibers and a diffuse vimentin network. Remarkably, five days IL-15 treatment (10 pg/mL) prevents RPTEC from EMT commitment since these cells maintain their initial epithelial-like morphology (basal d0), characterized by the expression of the epithelial markers CK and ZO-1 and the absence of detectable α-SMA and vimentin networks. By contrast, five days of rhIL-15 treatment (10 pg/mL) induced the opposite effects on RCC, causing not only the decrease of E-cadherin on RCC (Figure 4A), but also favoring the acquisition of a mesenchymal-like phenotype, as shown by the loss of epithelial markers (cytokeratins and ZO-1) and by the enhancement of vimentin and α-SMA stress fibers networks (Figure 7A).

In the light of these results, it was interesting to find out whether the reestablishment of the IL-2Rγ chain-dependent signal transduction pathway in RCC could interfere with the rhIL-15-induced epithelial-myofibroblast transdifferentiation process. Flow cytometry (Figure 7B) shows that the introduction of IL-2Rγ chain or JAK3 does not modify cytokeratins and vimentin expressions on untreated and rhIL-15-treated RCC (data not shown). In agreement with the data observed on E-cadherin expression (Figure 6C), only the IL-2Rγ/JAK3 co-transfection in RCC inhibits the cytokeratins down-regulation and vimentin upregulation observed after 48 hours of rhIL-15 treatment. It should be emphasized that the reestablishment of the IL-2Rγ chain-dependent signal transduction pathway in RCC, inhibits the rhIL-15-induced EMT process preserving the E-cadherin expression.

## Discussion

The clear cell renal cell carcinoma (RCC) is one of the most resistant solid tumors to chemo-and radiotherapy. The modest results obtained in the treatment with IL-2 and IFN-α require the development of new immuno-therapies. In this context, IL-15, which is currently used in clinical trials for the treatment of kidney cancer (NCT01021059 Protocol) [2] could be an useful alternative based on its immuno-activation activities [43]. However, it is important to be aware of the potential side effects of IL-15 on renal epithelial cells, especially tumor cells since until now the IL-15 action in renal physio-pathology is still not completely understood [20], [44], [45]. In this context, studies in IL-15 (IL-15−/−) and IL-15Rα (IL-15Rα−/−) deficient mice indicate that intrarenal IL-15 is an autocrine anti-apoptotic factor for renal tubular epithelial cells [20], [22], highlighting the central role of IL-15 and IL15Rα chain in renal homeostasis as survival factors. However, the role of the other IL-15R subunits and namely of IL-2Rγ (CD132) in the kidney is yet to be determined. Moreover, IL-15 has been found to participate in the development of solid tumors [43] notably, in renal carcinoma where, stimulation of the membrane-bound IL-15 by soluble IL-15Rα chain favors epithelial to mesenchymal transition [29]. Therefore, IL-15 application in tumor therapy should always be approached with caution and should be preceded by a careful examination of its effects in the appropriate tumor cells *in vitro* [43]. Taken together, these observations led us to reassess the role of IL-15 in primary cultures of human tubular epithelial renal cells of normal (RPTEC) and tumoral (RCC) origin.

As shown previously, our data show that primary RPTEC express a functional heterotrimeric IL-15Rαβγ complex whose stimulation with physiological concentrations of rhIL-15 (10 pg/mL) activated signaling pathways dependent on the IL-15Rα chain (IκBα), IL-2Rβ chain (MAPK-ERK1/2) [46] and IL-2Rγ chain (STAT5) [6], [8]. In a model where the deprivation of corticosteroids, together with absence of daily medium change, induces within five days in RPTEC an epithelial-mesenchymal transition (EMT), we show that addition of physiologic concentrations of rhIL-15 is sufficient to inhibit EMT commitment preserving E-cadherin expression, a main component of the adherent junctions and a master programmer of the EMT process [39], [40], [41]. Furthermore, the up-regulation of E-cadherin expression by rhIL-15 in RPTEC is dependent on the γc chain-signaling pathway as shown by the use of neutralizing anti-γc mAb and specific inhibitors for JAK3 and STAT5. Our data show that IL-15 is not only a survival factor for epithelial cells but also can preserve through the γc-signaling pathway, renal epithelial phenotype demonstrating that the cytokine possess a wide range of actions in epithelial homeostasis, as already shown for other tissues [25].

The major feature distinguishing primary normal epithelial renal tubular cells from those derived from clear cells renal adenocarcinomas is represented by the differential expression of the γc chain and of its cognate kinase JAK3 both *in vitro* and in tissue specimens derived from normal kidneys and renal clear cells adenocarcinomas. Indeed, primary RCC are characterized by the loss of the γc chain both at the transcriptional and protein levels, by a weak expression of JAK3 transcripts and by the lack of the functional 116 Kda JAK3 [47]. Immunohistochemical analysis on tissue specimens from renal clear cell adenocarcinomas highlights in comparison with normal counterparts the absence of γc chain expression and a strong decrease of JAK3 expression (−70%).

Scatchard's plot analysis on RCC reveals the presence at the cell surface of a single class of high affinity receptors (Kd = 375 pM, 413 IL-15 binding sites per cell). The specific IL-15 binding, which was completely abrogated by neutralizing antibody against the IL-2Rβ but not the γc chain, suggested the presence on RCC of an IL-15Rα/IL-2Rβ complex, whose existence was confirmed performing co-immunoprecipitation experiments. Previous data on γc^−/−^ knockout mice reported the existence of IL-15Rαβ heterodimers able to induce IL-15 endocytosis, but without demonstrating its capacity to induce signal transduction [36].

Indeed, stimulation of RCC with physiological concentrations of rhIL-15 triggers the signal dependent on the IL-15Rα (IκBα), the IL-2Rβ chain (MAPK-ERK1/2) [46], but not on the γc chain (STAT5) [6], [8]. The phosphorylation of ERK1/2 is a key-signaling event for the induction of the EMT process in response to several different inducers [48], [49], [50], [51], [52], [53]. This IL-15 signal in RCC induces the loss of E-cadherin expression [54], and favors the loss of the epithelial phenotype leading to the acquisition of a migratory one [39], [40], [41]. This hypothesis is supported by the results of transfection experiments showing that transient co-expression of γc chain and JAK3, necessary to reestablish the γc chain-dependent IL-15 signaling (phosphorylation of STAT5), counterbalances the IL-15 effects on RCC, inhibiting EMT process. Indeed, rhIL-15 preserved on γc/JAK3 co-transfected RCC the expression of epithelial markers (E-cadherin and cytokeratins) inhibiting the up-regulation of mesenchymal markers as vimentin.

Transfection of JAK3 or the γc chain alone in RCC is not sufficient to reestablish the γc chain-dependent IL-15 signaling (phosphorylation of STAT5), which is restored only after co-transfection of both molecules. These data demonstrate the existence in RCC of a double defect involving both the γc chain and the 116 kDa JAK3 isoform. The loss of expression of the IL-2Rγ/JAK3 couple in RCC is not induced by mutations [55], as observed in syndromes of severe combined immunodeficiency (SCID) [9] but probably related to different mechanisms of post transcriptional control targeting IL-2Rγ could involve its mRNA stability [56], while the loss of JAK3 expression *in vitro* and its weak expression *in vivo* may depend on the level of expression and activation of the tyrosine phosphatase SHP1, which negatively regulates JAK3 [57]. Alternatively, the residual expression of JAK3 in samples of renal cancer could be explained by the presence of splice variants of JAK3 lacking the kinase activity essentially detected in various human epithelial cancers cells [47].

These data highlight the central role of IL-15 γc-signaling in renal epithelial homeostasis and strengthen the fact that the loss of the γc chain in renal clear cell adenocarcinomas could represent a mechanism that through E-cadherin down-regulation not only favors EMT but may also affect trafficking, survival and functions of different subsets of CD8+ cells. In this respect, the potential role of the CD8+/CD103+ tumor infiltrating T lymphocytes (T-TILs), which exert powerful T cytotoxicity against tumor cells, through CD103/E-cadherin interactions is of particular interest [58], [59].

In conclusion, these data underline a novel role of IL-15, through the γc-signaling pathway, in the preservation or not of renal epithelial homeostasis according to the positive (RPTEC) or negative (RCC) regulation of E-cadherin expression. However, on the basis of these results it must be stated that at present there is no evidence that absence of γc/JAK3 is involved in the initiation of renal clear cell carcinoma.
